# Microbial fermentation as a strategy for the abatement of peanut allergens

**DOI:** 10.1128/aem.02497-25

**Published:** 2026-04-27

**Authors:** Nadia Yammoul Guzman, Christopher P. Mattison, John G. Gibbons

**Affiliations:** 1 Department of Food Science, College of Natural Sciences, University of Massachusetts Amherst 136235, Amherst, Massachusetts, USA; 2 Food Processing Sensory Quality, USDA Agricultural Research Service, New Orleans, Louisiana, USA; The Pennsylvania State University, University Park, Pennsylvania, USA

**Keywords:** Ara h, bioinformatics, epitopes, fermentation, immunoglobulin E, proteases, secretome

## Abstract

Although valued for their nutritional content and flavor, peanuts are ranked among the nine major food allergens in the United States, capable of inducing life-threatening reactions mediated by immunoglobulin E (IgE). Ara h 1, Ara h 2, Ara h 3, and Ara h 6 are recognized as major allergens due to the prevalence and structural stability of their epitopes, which make them resistant to digestion and processing. To address these challenges, processing treatments of different natures have been tested (e.g., physical, chemical, and biological) to reduce the allergenic content and create safer and more nutritious nuts. Allergen degradation studies discussed in this paper highlight the roles of endogenous peanut enzymes and microbial metabolites, as well as computational tools useful to model theoretical protein transformation under degradative treatments. Fermentation is emphasized for its multifactorial potential, combining the synthesis of bioactive metabolites by generally recognized as safe (GRAS) microbes with their proteolytic and redox activity to reduce allergenicity in peanuts while improving nutritional and sensory quality. Despite significant progress of these approaches, the development of a viable hypoallergenic peanut product remains elusive. Therefore, potential fermentation enhancers are discussed, including the addition of naturally extracted polyphenols that can alter the structure of peanut epitopes while increasing their proteolytic susceptibility. This review integrates structural insights, physical, chemical, and biological interventions, and computational approaches to provide a comprehensive perspective on peanut allergen reduction. By emphasizing epitope-targeted fermentation and synergistic treatments, we outline directions for developing safer peanut-based foods that balance efficacy, feasibility, and consumer acceptance.

## INTRODUCTION

Peanut allergy is a growing global health concern, affecting not only children but also an estimated 4.6 million adults in the United States. Classified as one of the nine major food allergens, peanuts have a balanced macronutrient profile, comprising 24%–26% protein, 47%–50% fat, and 15%–21% carbohydrates. Researchers have identified 18 allergenic proteins in peanuts, named Ara h 1 through Ara h 18 (1–3). These proteins primarily function as seed storage and structural components (4). Food allergic reactions (type I hypersensitivities) are specifically mediated by immunoglobulin E (IgE) antibodies (5). Currently, Ara h 1, Ara h 2, Ara h 3, and Ara h 6 are recognized as the major peanut allergens (6). Each allergen contains multiple IgE-binding epitopes, which can trigger immune responses ranging from mild symptoms like swelling and digestive discomfort to severe reactions such as airway obstruction, throat constriction, and life-threatening anaphylaxis (7).

Despite significant efforts, including the development of oral and topical immunotherapies, no effective treatment has been established to abolish peanut allergenic reactions. The adverse side effects of these therapies often lead patients to cease the treatments (8). Fermentation offers a promising biological approach to modulate allergenicity through multiple mechanisms. Early studies on legumes (1980s–2000s) screened allergenicity reductions of fermented soybean products, such as tempeh, tofu, and miso, while more recent investigations have evaluated fungal and bacterial fermentations in peanuts and other allergenic foods, alone or in combination with thermal and chemical treatments (9–12). Certain generally recognized as safe (GRAS) strains can hydrolyze allergenic proteins through secreted proteases and substrate adaptation, not only reducing allergenicity and toxin levels but also enhancing nutritional properties of the substrate (13). Modern computational resources can facilitate the rational design of targeted fermentation strategies by enabling *in silico* prediction of allergen hydrolytic sites and microbial enzyme secretion (14–16).

Not only do microbial enzymes participate in the amelioration of allergenicity through fermentation, but other metabolites, such as thiol-containing molecules and short-chain fatty acids (SCFAs), also contribute (17, 18). Thiol compounds have been shown to reduce disulfide bonds present in wheat allergens, which are also present in peanut allergens, thereby destabilizing their structure and increasing their susceptibility to proteolysis, while SCFAs play an important role in immune cell response modulation and the mitigation of adverse effects of sensitization to allergens (19, 20). Similarly, polyphenol extracts can interact with allergenic proteins through covalent and noncovalent bonding, decreasing IgE-binding capacity while providing antimicrobial benefits (21, 22).

This review focuses on the following ideas: (i) the structural properties of peanut allergens, (ii) results from fermentation-based studies on peanuts and related nut or seed allergen sources, (iii) the potential role of natural additives such as thiols and polyphenols in enhancing allergen reduction, and (iv) the role of modern *in silico* tools to guide future fermentation projects targeting allergens. Future perspectives integrating these approaches as a feasible path toward safer, consumer-acceptable hypoallergenic peanut products will also be outlined.

## STRUCTURAL BASIS OF PEANUT ALLERGENICITY

In legumes, many allergenic peptides are often derived from seed storage proteins that serve as energy reserves during germination and early seedling growth, while some also participate in plant defense mechanisms (23). Based on clinical trials and protein mapping studies, four major allergenic proteins in peanuts have been characterized: Ara h 1, Ara h 2, Ara h 3, and Ara h 6 (4, 24). Specific amino acid sequences of these proteins, called epitopes, can indicate the reactive potential of allergens in susceptible individuals, as they bind IgE and trigger subsequent immune responses. IgE epitopes can be classified as linear, a continuous sequence of amino acids, or conformational, having a discontinuous sequence of amino acids closely positioned by protein folding (25). Although their geometry within the allergen differs, conformational and linear epitopes are both relevant in triggering mild to severe clinical symptoms (e.g., anaphylaxis) from peanut consumption, with as few as eight amino acids capable of binding IgE and inducing histamine secretion by mast cells (24, 26).

**Fig 1:**
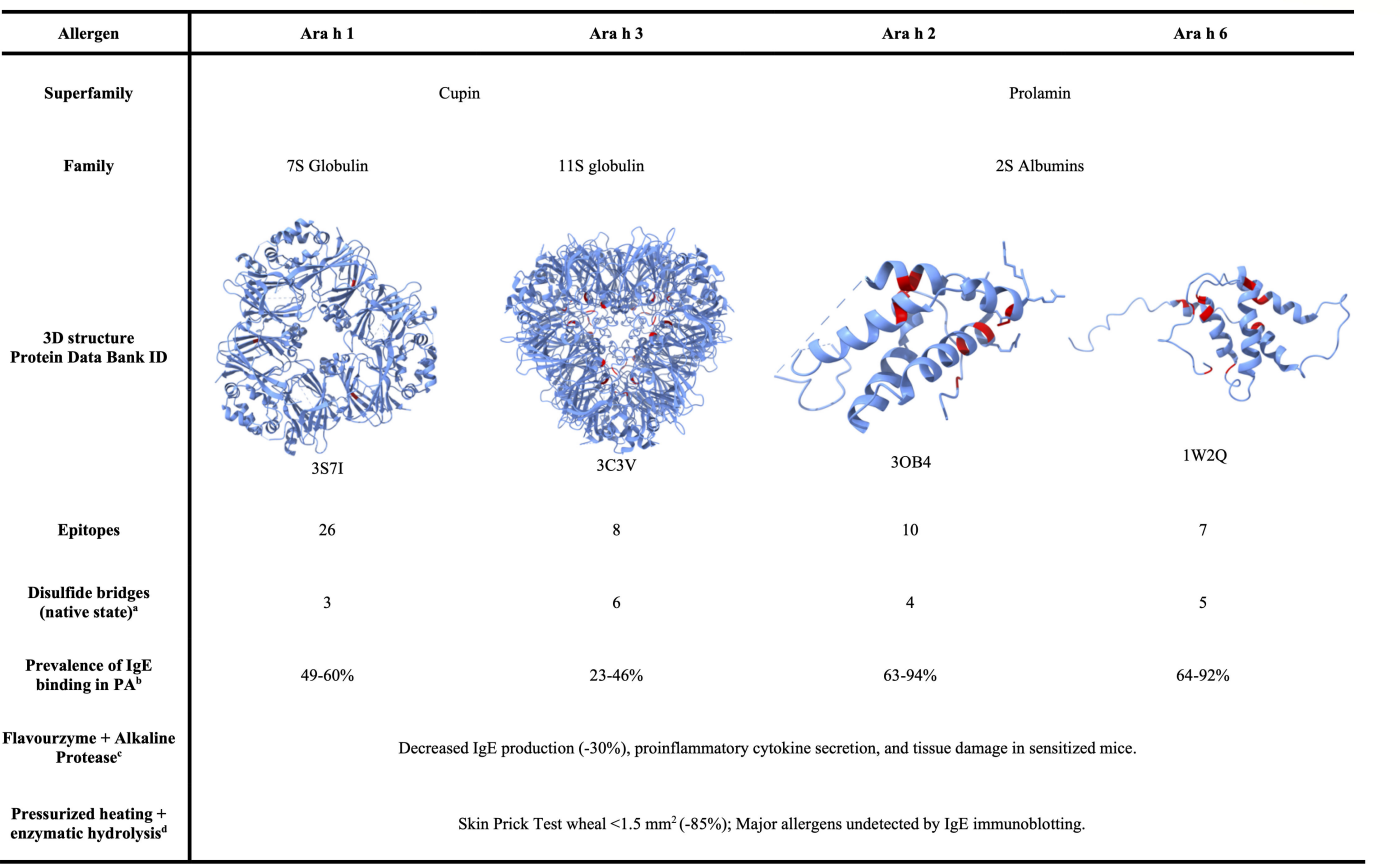
Structural and immunological characteristics of the major peanut allergens. The three-dimensional ribbon structures of peanut major allergens were visualized using UCSF ChimeraX (1.10) with cysteine residues highlighted in red (27). The published number of conformational and linear immunoglobulin E (IgE) binding epitopes for Ara h 1, Ara h 2, Ara h 3, and Ara h 6 is included; their specific amino acid sequences can be found in the Immune Epitope Database (IEDB) (4, 5, 25, 28). ^a^Ara h 1 forms a trimer, although conflicting reports exist regarding its disulfide connectivity (29, 30)*;* Ara h 3 forms a hexamer (6)*,* whereas Ara h 2 (4) and 6 are monomeric proteins in their native state (31). ^b^PA, peanut-allergic patients; *n =* 181 (32). ^c^*n=* 30; *-* indicates a reduction compared to untreated peanut controls (33). ^d^*n =* 27 (34).

Understanding the structural differences among Ara h 1, Ara h 2, Ara h 3, and Ara h 6, as well as the characteristics of their epitopes, is crucial for strategies aimed at reducing or eliminating peanut allergenicity. These four allergens are part of two protein superfamilies, cupins and prolamins, with distinct structural aspects, representing distinct challenges when modifying Ara h epitopes, as briefly summarized in Fig. 1. The cupin superfamily includes storage proteins in peanuts, including Ara h 1 (a 7S globulin/vicilin) and Ara h 3 (an 11S globulin/legumin). Ara h 1, recognized by 80% of peanut-allergic patients, is a soluble protein with a molecular weight of ~64 kDa (6, 26). Resistant to mammalian gut enzymes due to its globular hydrophobic structure with minimal exposed cleavage sites, Ara h 3 has a molecular weight of ~60 kDa and four linear epitopes (25). As reported by Becker, Petersen, and Jappe (6), Ara h 1 holds the highest number of total IgE linear and conformational epitopes; however, protein size and epitope number do not necessarily correlate with allergen structural stability, proteolytic resistance, or potency (5).

Ara h 2 and Ara h 6 weigh between 12 and 17 kDa, with fewer reported epitopes overall but requiring harsher treatments for disruption than the cupin allergens Ara h 1 and Ara h 3. As 2S albumins, the three-dimensional structure of Ara h 2 and Ara h 6 consists of α-helices stabilized by disulfide-bridged cysteine residues, four in Ara h 2 and five in Ara h 6, which are essential for allergenic activity and resistance to digestive enzymes (4, 31). Structural analyses show that their disulfide bridges remain intact after mild physical and chemical treatments, explaining their resilience to digestion and heat denaturation, thus making them reliable indicators for IgE-binding immunoassays in clinical trials (4, 24, 31). Some studies even report increased *in vivo* IgE binding in these two 2S albumins after high-heat treatments (e.g., frying, roasting), while others report equal or reduced binding compared to native Ara h (35–37). Proposed explanations include the formation of glycosylation end-products via Maillard reactions or protein unfolding that exposes previously hidden epitopes (35, 38).

The conformational diversity and epitope density of major peanut allergens make them difficult to disrupt with a single treatment. Enzymatic digestion alone represents a potential approach capable of cleaving allergenic epitopes but preserving T-cell–stimulating sequences. For instance, treatment of cashew storage proteins with pepsin, a key digestive enzyme found in the stomach, produced short peptide fragments (3–6 kDa) that markedly reduced allergenicity in sensitized mice, yet maintained their immunogenicity, showing efficacy as an alternative time-effective immunotherapy agent (39). Under controlled pre- and post-digestive conditions with pepsin (e.g., time, temperature, and enzyme concentration), major peanut allergens, Ara h 1, Ara h 2, Ara h 3, and Ara h 6 were screened for their capacity to activate basophils by β-hexosaminidase release (40). The results showed reductions of 3.2-fold for Ara h 1 and 5.9-fold for Ara h 3 after 60 minutes of pepsin digestion, compared to just 0.89- and 0.82-fold differences for Ara h 2 and Ara h 6, respectively (40). The same trend was noted after pepsin digestion, where reducing gel conditions showed hydrolyzed bands for Ara h 1 and Ara h 3, in contrast to unaffected bands of Ara h 2 and Ara h 6 across all time points (29). These findings support the concept that partial proteolysis can reduce IgE-binding capacity by direct cleavage of linear epitopes or through destruction of allergen conformation. However, complete mitigation of allergenic reactions may require a combination of treatments (e.g., physical + chemical; chemical + biological) to reduce IgE binding further and prevent histamine release not only from peanut consumption but from other major food allergens (41–43).

*In vivo* evidence for complete peanut allergen attenuation remains limited, but a research group recently tested the combined effect of pressure (physical) and enzymatic (chemical) treatments using skin-prick assays in 27 peanut-allergic individuals (see Fig. 1) (34). Their results, with pressure and enzymatically treated samples, showed wheal diameters of 1.5 mm^2^, which corresponds to a “low likelihood of severe allergic reaction” (category A, ≤2 mm) according to the Expert Panel criteria from the National Institute of Allergy and Infectious Diseases (44). Comparable findings were reported in hazelnuts, pistachios, and cashews when two food-grade enzymes were combined with heat and pressure by the same research group (45). The strongest hydrolytic effect was achieved with an Alcalase-like enzyme combined with high-pressure treatment, leading to a marked reduction in IgE binding in nut hydrolysates during skin-prick tests, although neither the identity nor structural details of the enzymes were disclosed (45). Complementary murine experiments further showed that treating peanuts with a combination of enzymes, a specific alkaline protease (details not specified) and the commercial enzyme blend Flavourzyme, resulted in significant reductions of allergic inflammation indicators (33). As described in Fig. 1, immunologically active proteins and mast cell degranulation in BALB/c mice significantly decreased compared to peanuts treated with one enzyme at a time and the control group. Also, the IC₅₀ for IgE inhibition increased from 0.0092 µg/mL (raw) to 0.172 µg/mL, indicating that approximately 19 times more protein was required to elicit equivalent IgE binding, reflecting weakened IgE hydrolysate interactions (33).

Proteases derived from plants and microbes, including trypsin, pepsin, α-chymotrypsin, papain, ficin, bromelain, Alcalase, Neutrase, and Flavourzyme, have been evaluated *in vitro* for their ability to disrupt major peanut allergens (Ara h 1, Ara h 2, Ara h 3, and Ara h 6) (39, 46, 47). Outcomes consistently demonstrate that, unlike the 2S albumins Ara h 2 and Ara h 6, the cupin proteins Ara h 1 and Ara h 3 are more readily disrupted by chemical and physical treatments. This greater susceptibility is attributed to their reliance on conformational epitopes, which are more prone to unfolding, aggregation, and masking. By contrast, the stabilizing disulfide bridges of Ara h 2 and Ara h 6, as described in Fig. 1, confer resistance and preserve their epitopes, making these albumins particularly challenging targets for chemical or physical disruption and subsequent allergenicity reduction without harsh treatments (25, 26, 29, 31, 47, 48).

## FERMENTATION AS A BIOLOGICAL STRATEGY

**Fig 2:**
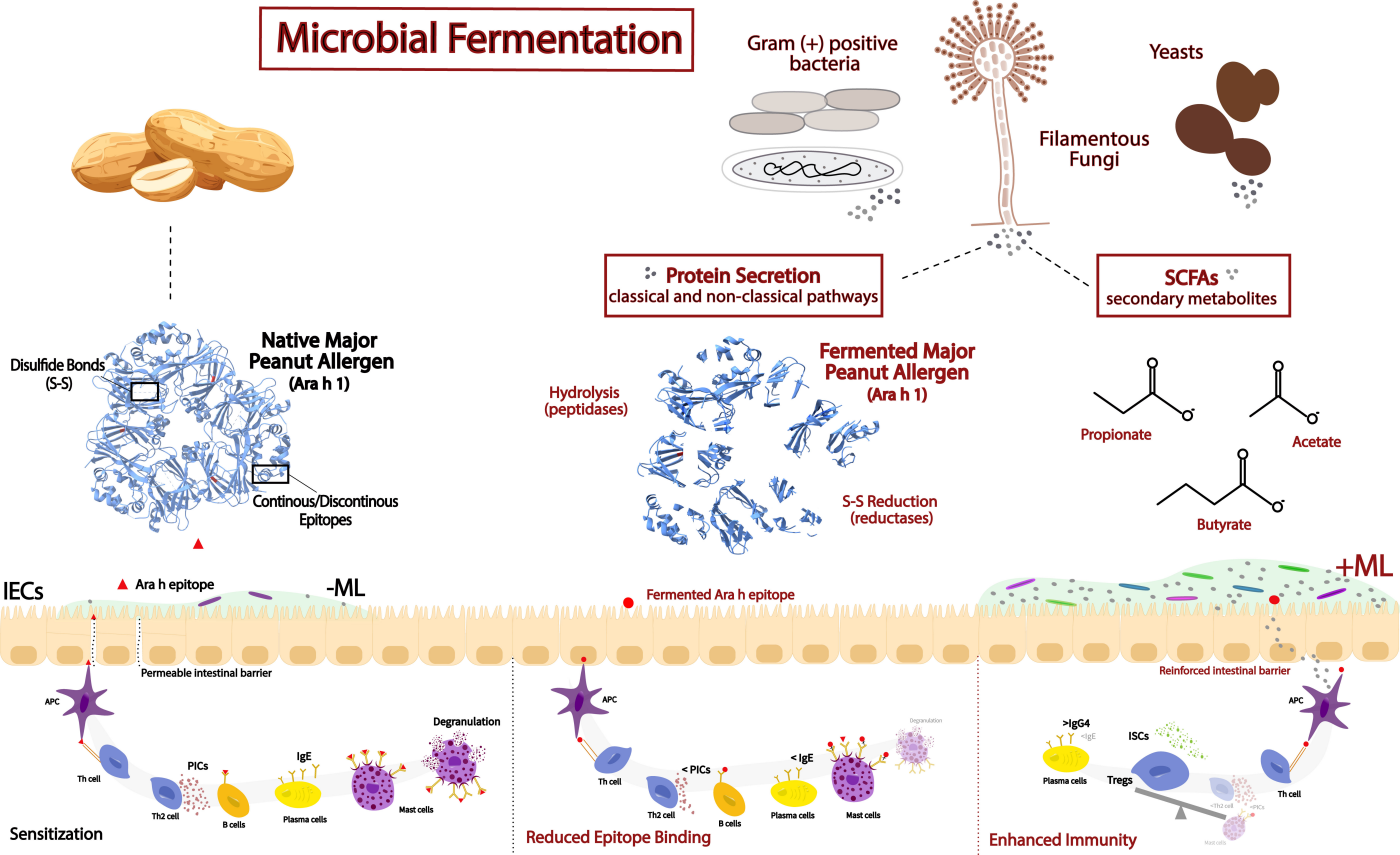
Simplified diagram illustrating the potential routes by which fermentation with GRAS microorganisms may mitigate or modulate allergenic responses to fermented peanut products. Fermentation-derived microbial enzymes and metabolites can alter allergenic proteins and influence immune pathways through effects on intestinal epithelial cells (IECs) and the mucosal lumen (ML). Microbial proteases and reductases secreted into the extracellular matrix (ECM) of peanuts modify the structural properties of allergens and their IgE-binding epitopes, thereby reducing their ability to trigger sensitization and histamine release. SCFAs reinforce the mucosal lumen (+ML) barrier and promote immune tolerance by modulating antigen-presenting cells (APCs) and T helper (Th) cell differentiation. The resulting immune balance favors regulatory T cells (Tregs) and inflammation-suppressive cytokines (ISCs) over pro-inflammatory cytokines (PICs), while enhancing the production of IgG4 “blocking” antibodies that counteract IgE-mediated responses.

Therapeutic approaches to suppress immune responses triggered by peanut epitopes have included oral immunotherapy (OIT) with defatted peanut flour (DPF), vaccines, and genetically modified peanuts, along with physical and chemical treatments mentioned in the previous section (6, 8). However, another avenue to develop potential therapeutic foods includes fermentation. Fermentation, originally an ancient practice to preserve foods, is now widely applied in different sectors for its role in the development of valuable metabolites produced by non-pathogenic fermentative microorganisms (49). In food systems, fermentation has gained increasing attention as a natural biological strategy to reduce allergenicity (41, 50, 51). During the fermentation process, molecules derived from both primary and secondary metabolism participate in altering the structure and allergenic potential of peanut allergens, as shown in Fig. 2. Beneficial molecules synthesized and secreted by GRAS fungi and gram-positive bacteria discussed in this paper include hydrolytic enzymes, organic acids, short-chain fatty acids (SCFAs), and antioxidants, which can enhance digestibility, vitamin availability, flavor, and immune-modulating capacity (20, 41, 52–57).

After the peanut seed matures, major allergens Ara h 1, Ara h 2, Ara h 3, and Ara h 6 are enclosed in rigid intracellular compartments of the cells called protein bodies, but progressive changes in cell wall structures during environmental stressors (e.g., pH, temperature, microbial activity) make them susceptible to proteolytic degradation (10, 58). Endogenous peanut exopeptidases and endopeptidases, produced during seed development and germination, have been shown to degrade storage proteins into soluble peptides and free amino acids that fuel seedling growth (23). Notably, one study examining Ara h 1 across a pH range of 2–10 found that at pH 3, aspartic endopeptidases in peanuts hydrolyzed IgE-binding epitopes of this major peanut allergen; although other peanut allergens were not screened, they may likewise be susceptible (52). Comparable studies of other peanut allergens remain limited. On the other hand, studies on cereals have also shown the activity of endogenous aspartic and cysteine proteases in degrading proteins stabilized by disulfide bonds, often activated by pH changes and capable of modifying conformational IgE-binding regions of wheat proteins (17, 59). These findings establish the potential role of endogenous proteases, pH, and redox potential in allergen degradation under optimal conditions.

The decrease of food allergen strength through fermentation is driven by the microbial secretome. The secretome can be defined as the complete collection of proteins released into the extracellular matrix by a cell, tissue, or organ under specific conditions and at a given time (60). Since microbes lack the ability to consume high-molecular-weight materials directly, their secretome encompasses an array of hydrolytic enzymes, particularly proteases, that degrade proteins into shorter peptides and thereby disrupting epitopes in major food allergens and decreasing the likelihood of allergic reactions, as shown in Fig. 2 (41, 61, 62). This enzymatic activity is often enhanced by primary metabolites (organic acids) synthesized by some lactic acid bacteria strains that create acidic environments and promote protein unfolding, as described previously.

The activity of bacterial and fungal proteases secreted during fermentation is dependent upon conditions, including pH, temperature, nutrient sources, metal ions, salinity, inoculum density, aeration, and fermentation duration (time) (13, 63). Specific preferences for temperature, nutrients, and ions in commonly used fungi, yeasts, and bacteria have been well described in several review papers (41, 63–65). Once released into the extracellular matrix (ECM) during fermentation, these enzymes initiate allergen unfolding and hydrolysis via the secretome of food-grade microbes (10, 45, 56). Table 1 summarizes some of the results from studies exploring the effect of different fermentation matrices (liquid, solid, semi-solid) on the modification of Ara h 1, Ara h 2, Ara h 3, and Ara h 6, and other selected nut/seed allergens. These experiments commonly use DPF or isolated protein fractions as substrates to test the specific cocktail of strains to minimize matrix interference while maintaining allergenic potential. It is worth mentioning that DPF is the active component in the FDA-approved oral immunotherapy formulation Palforzia (8). DPF retains allergenicity comparable to whole peanuts but with reduced matrix interference and less manipulation than the experimentally isolated protein fraction, which is known to induce changes in the IgE capacity of peanut allergens (35, 36, 46, 66).

**TABLE 1:** Effect of microbial fermentation on the IgE-binding capacity of major peanut allergens (Ara h 1, Ara h 2, Ara h 3, and Ara h 6) and food allergens from related legumes and nuts

Food matrix	Microorganism(s)	Fermentation time (h)	Detection methods^*b*^	Allergen	IgE-binding reduction (%)	Reference
Peanut pulp^*a*^	*Bacillus natto*	60	Indirect ELISA	Peanut allergens	77.3	(67)
Crushed peanut^*a*^	*Bacillus natto* and *Lactobacillus plantarum*	36	Indirect ELISA	Peanut allergens	90.2	(11)
Peanut pulp^*a*^	*Lactobacillus plantarum P1* and *Lactobacillus salivarius C24*	24	Sandwich ELISA	Ara h 1	74.7	(68)
Defatted peanut flour	Tempeh starter (*Rhizopus oryzae*)	48	Direct ELISA	Ara h 1, Ara h 2	~99, 80	(10)
Peanut milk^*a*^	*Lactobacillus plantarum* strain P1 and *Lactobacillus pentosus* strain Y6	24	Sandwich ELISA	Ara h 1	74.5	(69)
Whole cashews	*Rejuvelac culture (Pediococcus, Weissella,* and *Lactobacillus* spp.)	48	Cashew ELISA kit	Major cashew allergens (Ana o 1, Ana o 2, and Ana o 3)	66	(70)
Cashewgurt (commercial cashew yogurt)	*Lactobacillus* spp*., Bifidobacteria* spp*., Streptococcus thermophilus*	–^*c*^	Direct ELISA (human sera)	Cashew allergens	81	(71)
Defatted soy flour^*a*^	*Bifidobacterium breve*	48	Inhibition ELISA	Major soy allergen (Gly m 5)	100	(72)
Soy protein isolate	*Lactobacillus helveticus*	48	Sandwich ELISA	Major soy allergen (Gly m 5)	100	(50)
Tempeh (whole soybeans)^*a*^	*Rhizopus oligosporus* and *Actinomucor elegans*	48	Sandwich ELISA kit	Major soy allergens (Gly m 6, Gly m 5)	90.8	(73)
Soy Gly m 5 isolate	*Lactiplantibacillus plantarum* B1-6	–	Sandwich ELISA kit	Major soy allergen (Gly m 5)	95	(74)
Lupreme (commercial white lupin product)	*Rhizopus oligosporus*	22-26	LC–MRM–MS and *in silico* mapping of allergenic peptides	50% of allergenic peptides showed significant changes	–	(75)

^*a*^ Heat pretreatment.

^*b*^ ELISA, enzyme-linked immunosorbent assay; LC-MS-MRM, liquid chromatography-tandem mass spectrometry with multiple reaction monitoring.

^*c*^ –, detail not described in the paper.

Among promising studies described in Table 1, the fermentation of peanuts with *Bacillus natto* in submerged fermentation (SmF) or in combination with *Lactobacillus plantarum* in solid-state fermentation (SSF), the latter showed a greater reduction in IgE-binding activity, assessed by ELISA, of 90.3% compared to the *B. natto*-only SmF of 77.3% (11, 67). Another study fermented peanut flour with a commercially available fungal strain commonly used in tempeh fermentation and observed only slightly reduced IgE allergen binding (10). In soybeans, similar findings were demonstrated by a research group that was able to reduce IgE reactivity of major soybean allergens (glycinin, β-conglycinin) by ~90% in a solid-state fermentation with both *Rhizopus oligosporus* and *Actinomucor elegans*, *in vitro* (73). Although these studies demonstrate the potential of fermentation to reduce allergenicity *in vitro*, they share common limitations. Notably, immunological assays such as ELISA lack antibody specificity, limiting interpretation of which epitopes are degraded or which enzymes are potentially responsible. Moreover, *in vivo* data confirming the clinical relevance of these reductions in peanuts remain unavailable. Still, the data summarized in Table 1 and protease production investigations reveal that SSF, using minimal free water, often enhances extracellular protease secretions compared with submerged fermentation in filamentous fungi, yeasts, and lactic acid bacteria (13, 63, 64). In accordance with a direct comparison of *Bacillus subtilis* cultures, where a protease yield of 120 u/mL in SSF (960 U/g) versus 12 U/mL was reported in the submerged culture (76).

### Omics insights

Despite these limitations, recent omics data have provided molecular-level insights into how fermentation alters allergenic protein structures by integrating genomic, transcriptomic, and proteomic approaches. At the genetic level, several genes in both fungal and bacterial strains have been linked to the regulation or expression of protease activity (16, 49, 77). In fungi, an RNA-seq analysis of *Aspergillus niger* grown on peanut and cashew nut flours revealed over 2,400 genes upregulated compared to glucose controls, including known proteases such as oryzin and aspergillopepsin, as well as uncharacterized peptidases (54). These findings highlight the strong inducible capacity of fungal secretomes in response to nut-derived substrates. Recently in a genomic analysis, the *prtR* gene in *Aspergillus oryzae* was disrupted, causing a significant reduction in extracellular protease activity at several nitrogen sources, suggesting that *prtR* is a central transcriptional regulator of both endopeptidases and carboxypeptidases (78). Proteomic analyses of fermented peanuts with *Rhizopus oryzae* further identified time-dependent degradation of Ara h proteins and reduced antibody binding to Ara h 2, although reductions in IgE binding from allergic volunteers were minimal. The identification of *Rhizopus* proteins such as pyruvate carboxylase, heat shock protein, and aldehyde dehydrogenase suggests active microbial metabolism and secondary metabolite production may play a complementary role in allergen degradation (10).

In bacteria, Sieuwerts et al. (2011) investigated the transcriptomic effects of coculturing *Streptococcus thermophilus* and *Lactobacillus bulgaricus* in yogurt. Relative to the monoculture control, the combined culture of both strains showed higher expression of *L. bulgaricus prtB* and *S. thermophilus pepN*, genes that encode secreted peptidases (79). Similarly, integrative proteomic and structural modeling analyses demonstrated that the terminal pH achieved during LAB fermentation regulates protein folding and aggregation, thereby influencing the exposure and susceptibility of allergenic epitopes to gastrointestinal digestion (51). Soy protein isolates were fermented at controlled terminal pH values between 5.0 and 4.0, which resulted in the protein forming dense gel networks that promoted extensive degradation of epitopes with α-helix, random coil, and β-sheet structures, particularly in major allergens such as glycinin, β-conglycinin, P34, and basic 7S globulin. This effect was linked to the disruption of β-sheet conformational constraints, facilitating proteolytic access to buried cleavage sites and resulting in reduced immunoreactivity and improved protein digestibility (51). Together, these omics reports provide direct experimental evidence of how fermentation parameters shape microbial enzyme expression and substrate modification, valuable information that can be leveraged to develop more targeted hypotheses for allergen mitigation experiments.

### Immunomodulatory effects and mechanisms

Beyond modifying the structure of Ara h peptides, primary and secondary metabolites from fermentative microorganisms have been shown to play a significant role in decreasing allergenicity by exerting immunomodulatory and redox mechanisms (18, 68). Short-chain fatty acids (e.g., butyrate, propionate, acetate) and peptides generated during probiotic bacterial fermentation have consistently been shown to shift immune responses away from T helper 2 (Th2)-mediated allergic inflammation toward T helper 1 (Th1)-driven tolerance by inducing key cytokines that promote cellular immunity while suppressing Th2-associated IgE production (18). The underlying mechanisms by which SCFAs exert their immunomodulatory effects vary. They serve as substrates for mucosal immune cells and colonocytes and also inhibit histone deacetylases (HDACs), leading to increased histone acetylation and epigenetic regulation of immune-related genes. Notably, butyrate has been shown to act directly on naïve CD4+ T cells in a concentration-dependent manner. At low ranges, between 0.1 and 0.5 mM, butyrate enhances differentiation of naïve CD4+ T cells into Foxp3+ regulatory T cells (Tregs), which synthesize IL-10 and TGF-β, which are essential for maintaining oral tolerance to dietary antigens (80). In contrast, at higher concentrations (~1 mM), butyrate increases expression of the Th1 master transcription factor, T-bet, and promotes IFN-γ production and Th1 differentiation via HDAC inhibition (80). At the antigen-presenting cell (APC) level, butyrate reduces co-stimulatory molecules (CD80/CD86) on dendritic cells (DCs), promoting tolerogenic phenotypes, while enhancing retinaldehyde dehydrogenase (RALDH) expression in CD103^+^ DCs. RALDH converts vitamin A into retinoic acid, a key mediator for inducing Tregs and oral tolerance (18). In the context of food allergy, which is driven by Th2 cells secreting IL-4/IL-5/IL-13 and by IgE class switching, these immune cells shift induced by butyrate provide a mechanistic explanation for the Th1/Th2 modulations observed with fermented allergens.

A multi-omics study reinforced the connection between LAB metabolism and food allergen desensitization for a major pollen allergen, Bet v 1. LAB-fermented bee pollen proteins significantly reduced IgE and histamine levels in sensitized mice compared to the unfermented group and lowered mast cell infiltration in spleen and thymus tissues, alleviating allergic inflammation (81). Comparable results in gnotobiotic mice exposed to Bet v 1 were reported, where colonization with a mixture of *Lactobacillus* strains significantly reduced allergen-specific IgE and IgGs and increased TGF-β, alongside improved intestinal barrier integrity compared with germ-free animals (82). In this context, LAB fermentation of allergenic substrates acts externally to modify protein structure, whereas the presence of LAB within the gut mucosa acts internally, through their metabolites, to regulate immune balance and reinforce mucosal tolerance. A recent SSF co-culture fermentation of grain by-products (rice and wheat) with two filamentous fungal strains, *Aspergillus kawachii* and *Rhizopus oryzae,* was investigated to assess the reduction of common food allergy indicators in a murine model (19). The study combined histological, serological, and cytokine analyses, measuring splenic cytokine secretion (IL-4, IFN-γ, IL-17, IL-10, TGF-β), serum IgE and IgG by ELISA, and intestinal histopathology. Soluble fermented extracts significantly reduced footpad edema and inflammatory infiltration, increased the regulatory cytokines IL-10 and TGF-β, which can suppress hypersensitivity and inflammation, and decreased IL-4, IFN-γ, and IL-17, indicating activation of regulatory T cells (Tregs) and suppression of Th2- and Th17-driven allergic inflammation without altering IgE levels, suggesting immunomodulation rather than desensitization. Similar indicators were measured in a peanut study, where the diet of peanut allergic mice (*n* = 10) was supplemented with 0.5 to 1% ImmuBalance, a probiotic composed of fermented soybeans with lactic acid bacteria and the filamentous fungus *Aspergillus oryzae* (83). The highest dose (1% ImmuBalance) showed the most promising results, a ~60% reduction in peanut-specific IgE levels and an ~82% reduction in plasma histamine levels. These reductions were accompanied by a shift in cytokine balance, with decreased Th2-associated cytokines (IL-4, IL-5, and IL-13) and increased Th1 cytokine (IFN-γ), consistent with Th1/Th2 rebalancing (83). Together, these observations corroborate the hypothesis that fermentation-derived metabolites and bioactive peptides may enable shifts in gut microbial ecology that act synergistically to improve host immune responses, reduce allergic sensitization, and promote oral peanut tolerance.

### Fermentation enhancers

To increase the susceptibility of peanut allergens stabilized by S–S bonds to disruption, the fermentation environment can be shaped to favor redox mechanisms, as proteases alone may not efficiently degrade tightly folded allergenic peanut proteins, whose structural features both confer digestion resistance and IgE-binding capacity (24, 31, 55). Ubiquitous across all domains of life, the thioredoxin (Trx) and glutaredoxin (Grx) pathways catalyze the reduction of disulfide bonds in substrate proteins through thiol–disulfide exchange reactions (84). In these systems, Trx and Grx act as terminal reductases, while their recycling depends on NADPH-linked enzymes such as thioredoxin reductase and glutathione reductase, respectively (85, 86). Trx reduces protein disulfides via its active-site cysteine residues and is regenerated by thioredoxin reductase in an NADPH-dependent cycle (55). Similarly, the Grx system employs glutathione (GSH) as a redox cofactor, which acts as a broad-spectrum reducing buffer capable of destabilizing disulfide-rich proteins through its reversible oxidation to glutathione disulfide (GSSG) (20). While not a microbial study, a glutathione S-transferase (GST) was observed at increased levels in corn earworm (*Helicoverpa zea*) fed with cashew flour, the authors interpreted this upregulation as a possible response to the presence of disulfide-rich seed storage proteins in cashew nuts (84). These redox pathways are likely relevant to fermentation-based strategies aimed at increasing the susceptibility of disulfide-rich allergens.

*Lactobacilli* and certain yeasts are also known to produce and secrete Trx-based reducing systems, while LAB strains synthesize glutathione reductase, both systems playing a crucial role in the reduction of disulfide bonds in gluten present in wheat doughs (17, 53). When fermentation is combined with extracts rich in these reductases, disulfide bonds can be reduced, exposing previously inaccessible peptide regions, thus enhancing enzymatic hydrolysis. Though evidence for the reduction of peanut protein allergenicity remains scarce, Trx-enriched *Saccharomyces cerevisiae* enhanced ovomucoid proteolysis and reduced allergic symptoms in animal models, while rice-derived isoforms of Trx improved β-lactoglobulin digestibility and decreased immunoreactivity (20, 55). In sourdough systems, the role of the reducing agent GSH, produced by both *Fructilactobacillus sanfranciscensis* (previously described as *Lactobacillus sanfranciscensis*) and yeasts commonly found in sourdough (e.g., *Saccharomyces cerevisiae*, *Candida milleri*), was emphasized in preventing gluten polymerization and improving solubility and digestibility (17, 87). In agreement, another wheat fermentation experiment led to a marked increase in free thiol groups that coincided with degradation of α-amylase/trypsin inhibitor tetramers and suppression of pro-inflammatory cytokines in monocytic cell assays, highlighting the centrality of thiol release in redox-mediated allergen attenuation during wheat fermentation (53). These findings provide a strong mechanistic basis, but direct experimental evidence for such activity on peanuts and their disulfide-bond allergens remains to be corroborated.

Another potential natural booster for traditional fermentation is the addition of natural polyphenol-rich extracts, which have been shown to decrease the immunoreactivity of peanut allergens (22, 88, 89). For instance, a recent mouse model by Sun et al. (90) highlighted the role of epicatechin, a polyphenol highly present in apples, when bonded to peanut proteins in reducing systemic anaphylaxis, modulating cytokine profiles, and altering the secondary structure of Ara h 1, as confirmed by circular dichroism, which revealed α-helix disruptions after epicatechin binding (90). Cranberry and blueberry-fortified peanut flour, both naturally rich in polyphenols, demonstrated similar outcomes, suppressing *in vivo* basophil and mast cell degranulation and reductions in plasma IgE (88). This group also reported, for the first time, a dose-dependent decrease in CD63 expression, a key protein used as a marker of basophil activation. Simulated gastric digestion studies by Plundrich et al. (22) extended these findings, showing that Ara h 1, Ara h 2, and Ara h 3 incubated with cranberry and green tea solutions experienced higher destabilization during simulated pepsin digestion compared to controls. Their conclusions suggest that conformational changes interfered with pepsin activity and/or epitope accessibility, highlighting how the naturally acidic pH of these juices may have facilitated both hydrolysis and antioxidant activity of the complexes (22, 91). However, most nut and legume fermentations targeting allergenicity have been performed in water-based systems where the initial pH is neutral or near neutral (92). Hence, a potential shift of the matrix to initially acidic conditions using polyphenol-rich extracts from fruits could further enhance hydrolysis while changing protein structures through covalent and non-covalent polyphenol–protein complexes, altering their immunoreactivity (e.g., epicatechin and quercetin) (90). In addition, they may reduce pathogenic microbial growth, as observed in polyphenol-rich dry fermented sausages (21).

Taken together, evidence from microbial fermentation metabolites and enzymes underscores the multifaceted strategies available to attenuate peanut allergenicity, providing a strong basis for future applications. Beyond testing *in vivo*, future fermentation applications should also delve further into matrix parameters and supplements to enhance this secretion of proteases, as well as disclose specific enzymatic activity responsible for these modifications to individual Ara h peptides.

## *IN SILICO* APPROACHES GUIDING EXPERIMENTAL STRATEGIES FOR ALLERGEN REDUCTION

Modern immunological and bioinformatic resources now enable the rational design of experiments aimed at mitigating food allergenicity. Historically, approaches to characterize allergenic proteins relied on costly, labor-intensive experiments to map linear IgE-binding epitopes of major food allergens. The acquisition of detailed structural knowledge of these allergens has been greatly accelerated by modern computational tools. Technologies such as AlphaFold now enable *in silico* modeling of key features, including intra- and intermolecular bonding, potential cross-reactivities, and physicochemical properties, while comprehensive allergen databases provide information on epitope types (B cell or T cell; linear or conformational), immunoglobulin binding profiles, and structural homologies (93, 94).

Over the last decade, *in silico* methods have increasingly complemented *in vivo* and *in vitro* allergenicity research, giving rise to allergen informatics, a subfield of immunoinformatics (95, 96). The Immune Epitope Database (IEDB) is one of the most comprehensive web-based tools for epitope prediction, surpassing databases such as the International Union of Immunological Societies (IUIS), with data classified by B- or T-cell type, IgE or IgG binding, and continuous or discontinuous structure and supporting literature and assays (28). These types of bioinformatic tools also facilitate cross-reactivity analysis. For instance, the Structural Database of Allergenic Proteins (SDAP 2.0) is a peer-reviewed, manually curated repository containing over 1,500 sequences and the Cross-React tool which identifies isoallergens and predicts potential IgE cross-reactivity based on property distance values and physicochemical characteristics (97).

A recent study by Djeghim et al. (98) combined a mouse model with computational tools to assess allergenicity in six peanut accessions and to evaluate Ara h 3 cross-reactivity with the soybean allergen Gly m. Their workflow integrated SDAP for cross-reactivity, Dali for structural homology, IEDB for epitope mapping, and ClusPro (PIPER) with PDBsum/PyMOL for IgE docking (98). Similarly, in 2016, Ramesh et al. applied NetMHCIIpan 2.0 to predict Ara h 1 T-cell epitopes binding to key immune response initiators and validated predictions with basophil degranulation assays, identifying several immunodominant peptide vaccine candidates (99). While both studies highlight the power of *in silico* approaches, they also stress the necessity of experimental validation.

Computational advances extend beyond allergenic protein structure to include enzymatic and chemical hydrolysis prediction (100–102). Tools such as PeptideCutter, Rapid Peptides Generator (RPG), and PROSPERous Plus allow theoretical cleavage analysis of any amino acid sequence, facilitating the screening of peanut allergens for hypothetical cleavage patterns (e.g., proteases), identifying specific sites within the protein sequence (see Table 2 for descriptions). In an attempt to test this theoretical cleavage across peanut allergens and their mapped epitopes, we screened the amino acid sequences of peanut major allergens Ara h 1, Ara h 2, Ara h 3, and Ara h 6 in PeptideCutter. The results listed 37 cleavage agents, including chemical molecules (e.g., formic acid) and proteases (data not provided). A similar strategy was applied by Ye et al. (103) to cow’s milk allergens, where the selection of proteases (trypsin, pepsin, alkaline protease, papain) was guided by PeptideCutter and validated *in vitro*, with the strongest epitope reduction achieved when one of the resulting proteases was combined with *Lactobacillus* fermentation (103).

**TABLE 2:** Bioinformatic tools supporting experimental design for allergen reduction^*a*^

Tool	Function	Version	Reference
IEDB	Database of experimentally validated immune epitopes (linear and conformational) also includes predictive tools	Continuously updated	(28)
NetMHCIIpan	Algorithm to predict MHC-II-binding peptides allows epitope mapping	2.0	(104)
SDAP 2.0	Database of allergenic proteins and diverse tools for IgE potential of amino acid sequences	Updated every 8 weeks	(97)
UniProt	Comprehensive protein database with information about enzymes (EC), sequences, structures, and functional classification	Continuously updated	(105)
MEROPS	Comprehensive database for proteases with information about inhibitors, structures, and amino acid sequences	Continuously updated	(106)
PeptideCutter (ExPASy Server)	Predicts cleavage sites of amino acid sequences by a curated list of proteases and chemicals	–^*b*^	(100)
Rapid Peptides Generator	Predicts cleavage sites of amino acid sequences by proteases	–	(101)
PROSPERousPlus	Prediction of protease-specific substrate (amino acid sequences) and their cleavage site	Continuously updated	(102)
eggNOG-mapper	Functional annotation of genes and proteins based on orthology	2.0	(107)
BLASTp	Protein-protein aligner of homologous sequences	2.14	(108)
SignalP	Prediction of classical protein secretion pathways	6.0	(109)
DeepLocPro	Prediction of prokaryotic protein subcellular location	1.0	(110)
DeepLoc	Prediction of eukaryotic protein subcellular location and membrane type	2.1	(111)

^*a*^ Peer-reviewed software and databases for protein structure analysis, cleavage site prediction, genome annotation, and screening of microbial secretome.

^*b*^ –, information not available.

**Fig 3:**
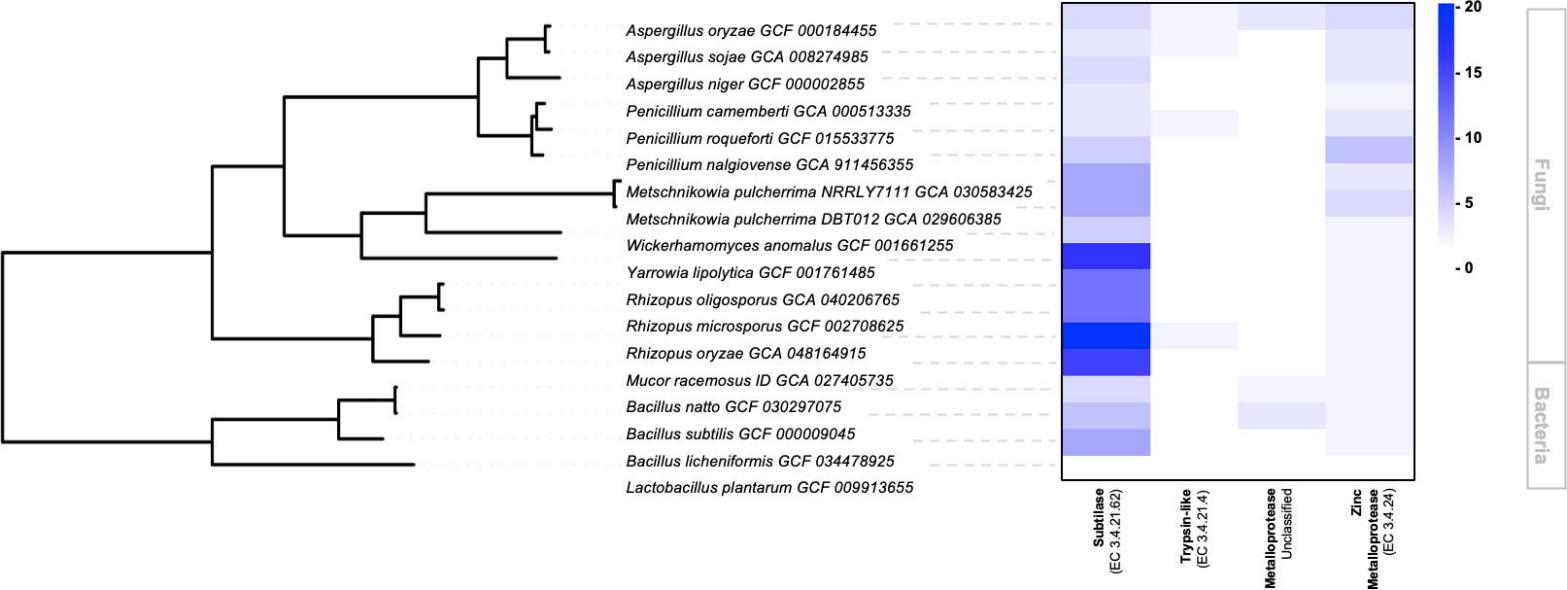
Phylogenetic and functional analysis of GRAS microorganisms associated with peanut allergen hydrolysis. Phylogenetic tree showing the potential evolutionary relationships of these species based on orthologous protein sequence groups. The tree was constructed using OrthoFinder with DIAMOND (112). Bootstrap values are of equal size, corresponding to a value of 1 (100%) for all nodes. Comparative secretion profiles of proteolytic enzymes across fungal and bacterial strains. Genome sequences and protein annotations were retrieved from the NCBI (National Center for Biotechnology Information), and individual protein annotations (when available) were analyzed using the BLASTp database. Secretory pathway screening was performed using SignalP6 and Secretome 2.0 (DeepLoc) to identify classical and non-classical secretion routes, respectively. EC, Enzyme Commission number (113).

After allergen amino acid sequences were screened in PeptideCutter to identify candidate cleavage enzymes of peanut major allergens, the resulting proteases were collected and used to query the genomes of GRAS microorganisms, thereby linking structural allergen data to proteases of the microbial secretome. Some of the strains screened in this work have been previously used to improve the sensory or nutritional aspects of foods, such as *Yarrowia*, *Penicillium*, *Mucor*, and *Metschnikowia* spp., but to our knowledge, remain unexplored in allergen catabolism analysis, while *Bacillus*, *Lactobacillus*, *Rhizopus* spp. have already been used to test allergenicity reductions (Fig. 3). The secretory pathways of bacteria and fungi species diverge significantly due to fundamental differences in cellular organization. Bacterial secretion involves direct, membrane-associated translocation across a single cell barrier, whereas fungal secretion relies on a complex, multi-step vesicular transport system supported by distinct organelles and extensive post-translational processing (62, 77). Still, both systems depend on N-terminal signal peptides to transport target proteins across membranes into the growth medium. This conserved feature enables the use of bioinformatic pipelines to expand and facilitate *in silico* secretome comparisons across prokaryotic and eukaryotic species using accessible resources, described in Table 2, such as SignalP6 and Secretome 2.0 (recently renamed Deep Loc and Deep Loc Pro), which predict classical and non-classical secretory pathways across organisms, respectively (109–111).

The data from the annotated proteins and secretome analysis with SignalP6 and Secretome 2.0 revealed a diverse repertoire of extracellular proteases among the different microorganisms screened. This output was used to construct a comparative heatmap linking theoretical allergen cleavage with the predicted protease repertoire of GRAS microorganisms, demonstrating the potential of current immuno-informatics and bioinformatics resources to support the design of experiments targeting allergen reduction through microbial fermentation in a more supported and efficient manner (see Fig. 3). The literature corroborated most differences in protease families secreted by the organisms screened in Fig. 3. A higher number of subtilases and metalloproteases was expected from *Penicillium*, *Yarrowia*, *Rhizopus*, *Aspergillus, Mucor,* and *Bacillus* species, and these peptidases play key roles in nutrient acquisition, and most have been previously studied for their effects on allergens or have been used in different industries for their proteolytic capacity (e.g., cheese, breads, soy products, acids, detergents) (13, 65, 114). *Wickerhamomyces anomalus* and *Metschnikowia pulcherrima* are used industrially to produce acids and bioethanol; however, subtilases are present in most fungi as they are required for nutrient acquisition (44). Surprisingly, *Lactobacillus plantarum* is not expected to secrete any of the peptidase families investigated, even though it has previously shown, alone and in co-fermentation, to reduce allergen content and activity, suggesting that Lactobacillus may play a role primarily in modulating immune system responses rather than directly through extracellular allergen proteolysis (11, 68, 69, 103).

To our knowledge, this heatmap represents the first integrative *in silico* workflow combining allergen cleavage prediction with GRAS protease mining, although these tools have independently been used to analyze prokaryotic and eukaryotic enzymatic systems (115, 116). Nevertheless, several limitations of *in silico* approaches must be recognized, including the structural complexity of Ara h allergens and the multifactorial nature of allergic reactions. Although secretome prediction allows rapid identification of potential secreted proteins, accurately modeling the full biological interactions within complex systems remains challenging. Moreover, a predicted secretory protein may not be expressed under the specific cellular or environmental conditions studied or may be post-transcriptionally regulated or retained within the cell. Thus, integrating computational results with multi-omics data can enhance the identification and validation of truly secreted proteins (15, 65, 117).

## FUTURE SCOPE AND CONCLUSIONS

Despite numerous advances in computational tools and the diverse array of experimental techniques designed to reduce or eliminate the IgE-binding ability of peanut proteins, the first allergen-free peanut product has not yet been recorded. Chemical and physical approaches to disrupt Ara h 1, Ara h 2, Ara h 3, and Ara h 6 and reduce their IgE-binding capacity have shown promising outcomes but have only achieved partial, rather than complete, allergen reduction. Among promising processing technologies to counteract allergic reactions in peanuts, fermentation has gained particular attention in recent years. Specifically, fermentation is a traditional process that employs beneficial microorganisms capable of metabolizing allergenic proteins while enhancing the nutritional, immunological, and sensory properties of the final product. Some of the most significant reductions in sensitization to major food allergens have been recorded through mixed-strain fermentations or monocultures combined with physical or chemical treatments. GRAS microorganisms commonly used in dairy, gluten, nut, and legume fermentations have not only demonstrated hydrolytic capacity but also the ability to regulate immune cells involved in sensitization, as discussed in this review.

Acting through structural modification, precipitation, enhanced proteolytic susceptibility, and immune modulation, polyphenols show strong potential as natural modulators of peanut allergenicity. Thus, fermentation with polyphenol-based or thiol-rich supplements could represent a synergistic biochemical strategy, where microbial metabolism under these conditions enhances allergen modification through protein-binding or disulfide bond reduction, increasing allergen disruption while potentially modulating immune responses (22, 53, 90). The application of these strategies to develop novel peanut products with reduced allergenicity remains at a preliminary stage, since specific dosages and methods for reproducibility, as well as federal regulations and consumer acceptance, remain to be investigated to incentivize large-scale production.

Experimental trials from strain screening to post-fermentation proteomics remain resource and time-intensive, but these limitations can be diminished through computational advances in biology and chemistry. Integrating immuno-informatics and molecular modeling software with multi-omics technologies can reveal how microorganisms and their secretomes (proteases, reductases) bind and alter specific epitope regions within peanut allergens. These mechanistic insights into allergen-enzyme binding interactions can be used to strengthen experimental designs for allergen reduction, such as engineering “precision fermentation” strains (e.g., CRISPR editing) that consistently produce high yields of molecules involved in peanut allergen reduction (118, 119). Such combined approaches, integrating *in silico* and wet-lab experimentation, may support advances in epitope-targeted fermentation, a concept potentially comparable to precision fermentation (115, 120).

This review has highlighted biochemical and computational strategies that complement fermentation-based approaches to mitigate IgE-mediated type I hypersensitivity to peanuts. Physical treatments were mentioned only briefly, as they lie beyond the primary scope of this discussion. Collectively, research on the structural complexity of peanut epitopes, natural enzymatic degradation, integrative biotechnological methods, and *in silico* approaches demonstrates strong potential for developing peanut products with reduced allergenicity in a more efficient manner. Moving forward, ensuring economic feasibility and consumer acceptance will be crucial for translating these advances into widely accessible hypoallergenic foods.

### Highlights

(i) Structural and IgE-epitope insights guide strategies for peanut allergen reduction. (ii) Solid-state coculture fermentation shows capacity for mitigating allergenicity. (iii) Computational tools enhance rational design of fermentation-based interventions. (iv) Natural enhancers like polyphenols may boost microbial allergen mitigation effects.
